# Interdisciplinary Research and Impact

**DOI:** 10.1002/gch2.201900020

**Published:** 2019-04-05

**Authors:** Rebekah Brown, Lara Werbeloff, Rob Raven

**Affiliations:** ^1^ Monash University VIC 3800 Australia; ^2^ Monash Sustainable Development Institute Monash University VIC 3800 Australia

## Background

In 2015, all countries in the world signed up to 17 Sustainable Development Goals (SDGs). The goals are ambitious for many reasons, not least because of the diversity of topics and challenges addressed through them. It is widely acknowledged that realizing the SDGs requires solutions‐oriented, interdisciplinary research capable of bridging traditional divides between disciplines and also combines research excellence with relevant impact.^1^, ^2^ However, so far there is only limited understanding of the enabling conditions, challenges, lessons, and tools for interdisciplinary sustainable development research. Furthermore, interdisciplinary research is often considered overly difficult, time‐consuming, hard to publish and challenging to get funded.

Increasing our understanding of how to effectively design and deliver interdisciplinary research is crucial to realizing the SDG agenda. This special issue is an early attempt to fill this gap by systematically bringing together and comparing experiences across six international, interdisciplinary sustainable development research collaborations that are seeking to influence policy and practice in their respective domains. We are particularly interested in understanding how such projects are set up and implemented, and then translate their findings to deliver real‐world impact.

## A Framework for Comparing Interdisciplinary Research and Impact

Comparing the experience of operationalizing interdisciplinary research across different projects requires a pragmatic framework. Here, we draw on five principles for interdisciplinary research^3^ initially conceived by Brown et al. (2015), drawing on the experience of growing an interdisciplinary water research program at Monash University in Melbourne, Australia.

### Principle 1: Forge a shared mission

This refers to developing a collective, compelling overall goal for the project, including impact as a necessary outcome, with meaningful roles across the different disciplines. A shared mission is considered important to maintain a sense of direction and purpose, which is particularly critical in the face of inevitable failures and setbacks likely to occur in ground‐breaking interdisciplinary projects.

### Principle 2: Develop t‐shaped researchers

T‐shaped researchers are researchers that cultivate deep disciplinary expertise, for example, by building disciplinary credibility through publication in the top journals in their field, while simultaneously fostering the ability to look beyond their own disciplines and appreciate the norms, theories, approaches, and breakthroughs in other disciplines.

### Principle 3: Nurture constructive dialogue

This principle refers to the need to develop the conditions and informal rules that empower researchers to engage across disciplines, which in practice is often challenging due to vast differences between disciplinary jargon. Core to a constructive dialogue is also fostering shared empathy, respect, and trust for alternative scientific approaches and learning to communicate effectively across disciplinary boundaries.

### Principle 4: Give institutional support

Interdisciplinary research is often challenging because of mismatches with disciplinary‐oriented academic careers. Promotion criteria, top‐journal publication lists that do not value interdisciplinary work, and faculties and school policies that inadvertently make collaboration across disciplines difficult all act as barriers. Institutional support, for instance through changes in university policies or interdisciplinary seed‐funding programs, is therefore critical.

### Principle 5: Bridge research, policy and practice

This refers to the active work required to create enduring connections between researchers, policy makers, and industry partners to build pathways for the adoption of interdisciplinary research outputs and ensure real‐world impact.

## Contributions to this Special Issue from the Perspective of the Five Principles

The contributions to this special issue cover a variety of empirical fields, geographical scopes, and disciplines (summarized in **Table** **1**). In their contribution, Jeff Waage and colleagues report on the experiences with the Leverhulme Centre for Integrative Research on Agriculture and Health (LCIRAH) in the UK, an initiative to integrate agriculture and health research communities for improved nutrition and health. The Centre was established in 2011 and grew into a diverse program of integrated research projects, attracting additional funding along the way and increasingly targeting and training a global community of interdisciplinary agri‐health professionals and researchers.

**Table 1:** Contributions to the special issue

				
				
				
				
				
				
				

Ana V. Diez Roux and colleagues describe the origins and characteristics of an interdisciplinary collaboration aimed at promoting and disseminating research for healthy cities in Latin America and the Caribbean. The collaboration was embedded in two initiatives: The Network for Urban Health and the Salud Urbana en América Latina project. The Collaborative Adaptation Research Initiative in Africa and Asia (CARIAA) project share insights from a seven‐year climate change adaptation program, with more than 450 researchers and practitioners involved across four consortia. In their contribution, Daniel Black and colleagues describe the development, conceptualization, and implementation of a research pilot, with the aim to explore how human and planetary health can become more central to urban development processes. David White and colleagues present and evaluate a sustainability research project in Pernambuca, Brazil, that aims to improve local capacity to manage existing and future water resources efficiently, sustainably, and equitably. Finally, Emily Nix and colleagues explore the use of participatory action research as a framework for facilitating collaboration with transdisciplinary researchers and with participant communities.

Comparing the experiences across the different contributions provides an opportunity to distil commonalities, challenges, lessons, and tools for each of the five principles identified by Brown et al. (2015).

### Principle 1: Forge a shared mission

Regarding this first principle, the papers share an emphasis on the importance of participatory processes to design goals, mission, aims, and approach, and develop a shared understanding. All papers describe a deliberate process of developing a shared problem framing, formulating common research questions and co‐creating research aims and approaches through extensive participatory processes.

For instance, in their contribution, Waage and colleagues demonstrate how the term “agri‐health” was deliberately created as a unifying label that could facilitate research across sectors and disciplines, enabling everyone to see their role. Collaborative designs of research questions and methods helped the integration of both disciplinary perspectives as well as perspectives of stakeholders on aims and methods and so on. Eventually, the efforts evolved in the co‐creation of a new discipline called “agri‐health” through a global training program. In seeking to forge a shared mission between interdisciplinary researchers and local communities, Nix and colleagues utilised participatory methods to allow participant perspectives to be integrated with the theoretical perspective of each discipline. This enabled a more bottom‐up approach to the cogeneration of theory, rather than imposing pre‐existing disciplinary theories onto other disciplines and community needs. White and colleagues show how a systematic regional water governance system analysis at the beginning of the program was primarily designed to contribute to a collaborative problem framing. Black and colleagues warn, however, that forging a shared mission between disciplines, academics, and the real world is a challenging process that requires a significant length of time, which should be taken into account when designing interdisciplinary programs. The development of momentum amongst participants is likely to be a foundational issue and fundamental to all other aspects of interdisciplinary research, according to Cundill and colleagues.

### Principle 2: Develop T‐shaped researchers

Developing T‐shaped researchers was also recognized in all contributions to be important for interdisciplinary research. The contributions highlight various mechanisms and tools for developing T‐shaped researchers, many of which come down to creating frequent opportunities for interactions across disciplinary teams and individuals. Waage and colleagues, for instance, report how to build upon a range of mechanisms, including co‐supervision of researchers by a minimum of two disciplines, focused interdisciplinary trainings to reach disciplinary specialists, learning labs to build interdisciplinary skills, and the deliberate attempts to co‐create shared conceptual frameworks to “exploit the integrative power of conceptual frameworks.” However, Waage et al. also note some of the challenges, such as student concerns about becoming “a jack of all trades – master of none”, in particular when disciplinary supervisors were lacking interdisciplinary expertise and skills. Roux and colleagues add that they deliberately remained open to including researchers not formally involved in the program and that they particularly fostered capacity for researchers at all levels, for instance via publication processes. Cundill and colleagues note the importance of team building and frameworks that support mutual leaning across disciplines, with an important role for “champions” – individual researchers that commit to a particular research topic and play a convening role. Nevertheless, despite willingness to overcome disciplinary boundaries, challenges often remain, as White and colleagues note in relation to conflicting methodological standards, and conflicts about the most appropriate tools to use. Eventually, a workable solution was found in establishing a mix of cutting‐edge research methods and practice‐oriented management models.

### Principle 3: Nurture constructive dialogue

Similarly, nurturing constructive dialogues across disciplines is enabled by sustained and structured interactions across disciplinary groups and individuals, and mobilizing a range of communication mechanisms. Regular team meetings, conferences, away days, shared work spaces, annual meetings, and cross‐institutional working groups are some of the examples mentioned by Waage et al. Ensuring time and funding is available to support regular in‐person interactions (for instance via annual meetings) is particularly important for those programs with international partners and collaborators. Nix et al. found that the iterative nature of interdisciplinary work takes time, requiring a greater degree of discussion and exchange with participant communities, as well as challenging the typically linear thinking of traditional disciplines. Although time consuming, that research team found these iterative discussions provide valuable flexibility and enhanced learning opportunities. To best support interdisciplinary collaboration among researchers, they recommend developing an effective communication framework, including elements such as reflections on implicit disciplinary discussions, vocabularies, cultural values and norms.

The contributions of Roux et al. and Cundill et al. reveal the value of developing clear principles and a statement of values to guide project collaboration and communication. Yet despite good intentions, there may remain challenges in nurturing constructive dialogues due to ‘decades of isolation’ between disciplines (Waage et al.) or systemic features such as pre‐existing norms and biases, formal rules laid down in partnership agreements, power asymmetries between partners geographical barriers and various layers of cultural competencies (Cundill et al.). White et al. found that government and scientific actors tended to dominate the research process while other interests, including environmental NGOs, agricultural interests, and marginalized populations, were underrepresented. In this respect, Cundill and colleagues as well as Black and colleagues argue for an important role for consortium coordinators, facilitators, champions, or knowledge intermediaries to play a key role in overcoming such challenges.

### Principle 4: Give institutional support

Building institutional capacity for interdisciplinary research is the fourth principle highlighted by Brown et al. (2015). Several contributions, including those by Cundill et al. and Waage et al., highlight the importance of having flexibility with the available funds to enable new, interdisciplinary collaboration that emerge during the process (beyond initial expectations), with a strong degree of open‐endedness. Black and colleagues highlight the benefits of a host institution that values expertise in applied research and real‐world interaction. Another important factor highlighted in the contributions by Black et al. and Cundill et al. is that interdisciplinary research requires more effort and resources for clear and transparent governance and leadership across disciplines, academic and other partners. Taking the time to set up systems for project management and administration from the outset emerged as a critical enabler. White and colleagues observe that differing academic outputs are needed and valued differently for researchers at various career stages, such as a PhD thesis, single‐author publications, management reports, or new model developments. Taking these differing requirements into account can help ensure researchers are willing and able to fully participate in an interdisciplinary collaboration.

### Principle 5: Bridge research, policy, and practice

Finally, the contributions to this special issue highlight the various mechanisms and ways in which bridging research, policy, and practice can be coordinated. In general, there is emphasis on the important role of diverse partners and networks in all areas of an interdisciplinary project. Waage et al. mention examples such as an advisory group providing external perspectives on the program, developing relationships with donors and researchers worldwide, and organizing conferences and meetings that include research sponsors and policy advisors. Roux et al. highlight the role of regional workshops with diverse stakeholders including governments, not‐for‐profit communities and academics, an explicit policy translation strategy with formal communication channels, and early engagement of stakeholders in the process through policy briefs. Nix et al. observe that issues of control and power over a research agenda may need to be navigated, requiring significant discussions to arrive at a shared understanding. In order to ensure interdisciplinary research is high quality, they also recommend developing a framework at the outset to assess validity of the research from the perspective of all the disciplines involved. White et al. show how they took an explicit transdisciplinary approach to work with a local stakeholder to solve real‐world problems. Some of the key project‐level tasks included defining the roles, responsibilities, and accountabilities for different actors involved, including discussing and balancing scientific rigor with societal relevance and practitioner capabilities. The latter is also regularly managed as a particular challenge in transdisciplinary research, as university researchers face expectations to publish in high‐impact journals that are mostly disciplinary‐oriented.

## Outlook

This special issue has started to explore the conditions, challenges, mechanisms, and benefits for interdisciplinary research to address grand societal challenges. The collection of papers crosses a large range of topics, all of them characterized by their specific histories, institutional contexts, participants, problem framings, and disciplinary engagements. The five principles help to look across these very diverse programs and start to distil some commonalities. While there is a long tradition of articulating the need for interdisciplinarity, we are still only standing at the beginning of developing a fundamental understanding of interdisciplinary research and how it operates in practice. Compared to disciplinary practice, interdisciplinary research so far remains under‐researched, under‐theorized, under‐developed, and under‐supported, and remains challenging in the light of mostly disciplinary‐oriented universities, publishers, and funders. We hope this special issue offers valuable insights into how interdisciplinary research collaborations can be operationalized to deliver real‐world solutions to entrenched sustainable development challenges.
